# Body Burdens of Polybrominated Diphenyl Ethers among Urban Anglers

**DOI:** 10.1289/ehp.8138

**Published:** 2005-08-08

**Authors:** Kimberly B. Morland, Philip J. Landrigan, Andreas Sjödin, Alayne K. Gobeille, Richard S. Jones, Ernest E. McGahee, Larry L. Needham, Donald G. Patterson

**Affiliations:** 1 Department of Community and Preventive Medicine, Mount Sinai School of Medicine, New York, New York, USA; 2 National Center for Environmental Health, Centers for Disease Control and Prevention, Atlanta, Georgia, USA

**Keywords:** fish consumption, PBDE, polybrominated diphenyl ethers

## Abstract

Polybrominated diphenyl ethers (PBDEs) have been widely used in the United States and worldwide as flame retardants. Recent PBDE production figures show that worldwide use has increased. To determine whether fish consumption is a source of PBDE exposure for humans, a cross-sectional epidemiologic study of New York and New Jersey urban anglers was conducted during the summers of 2001–2003. Frequency of local fish consumption was assessed by questionnaire, and blood samples for PBDE analysis were collected from 94 anglers fishing from piers on the lower Hudson River and Newark Bay. We analyzed PBDEs by gas chromatography–isotope dilution–high-resolution mass spectrometry. The congeners found in anglers’ serum at the highest concentrations were, by International Union of Pure and Applied Chemistry numbers, BDE-47, BDE-153, and BDE-99. Anglers reporting consumption of local fish had higher, but nonstatistically significantly different, concentrations of PBDEs than did anglers who did not eat local fish. For some congeners (BDE-100 and BDE-153), we observed moderate dose–response relationships between serum PBDE levels and frequency of reported fish intake. These findings suggest that consumption of locally caught fish is not a major route of human exposure for this study population.

Polybrominated diphenyl ethers (PBDEs) are a class of chemicals used extensively as flame retardants to decrease flammability of polymeric materials and textiles (Sjödin etal. 2003). PBDEs possess similar chemical and physical properties to polychlorinated biphenyls (PCBs), that is, high lipophilicity, low vapor pressure, and resistance to environmental degradation [World Health Organization (WHO) 1993, 1994]. Tetra- through hexa-BDEs have been shown to bioaccumulate, but an exception is decabromodiphenyl ether (BDE-209), which has been shown to have a surprisingly short half-life in people industrially exposed to this compound (Sjödin etal. 1999).

Three technical PBDE mixtures are produced and are identified by their average bromine content as pentabromodiphenyl ether (penta-BDE), octabromodiphenyl (octa-BDE), and decabromodiphenyl (deca-BDE). Penta-BDEs are used primarily in polyurethane foam, whereas octa-BDEs are used in acrylonitrile butadiene styrene (ABS) resin. Deca-BDE is used in a variety of polymeric materials (Alaee et al. 2003). PBDEs are additive flame retardants, and unlike reactive flame retardants such as tetrabromobisphenol A, they are not covalently bound to the polymer and therefore are more likely to leach out of the product during its lifetime (Sjödin etal. 2003).

Global production of PBDEs has increased rapidly over the past decade (deWit 2002; Ikonomou etal. 2002). The majority (95%) of the penta-BDEs are used in North America, and approximately 40% of global use of technical octa-BDEs and deca-BDEs occurs in North America (Bromine Science and Environmental Forum 2004). Manufacture and use of penta-BDEs and octa-BDEs have been discontinued in Japan and Europe, and voluntary withdrawal from the U.S. market took effect as of the end of 2004 (European Union 2003; Tullo 2003; Watanabe and Sakai 2001). However, manufacture and use of deca-BDEs are expected to continue indefinitely. Human and environmental biomonitoring will most likely be needed for years to come because of the high persistence and expected long half-lives of other PBDE congeners in environmental media.

PBDEs have been detected in sediments (Hassanin et al. 2004; Sellstrom et al. 1999; Tullo 2003; Watanabe and Tatsukawa 1989; Zegers etal. 2003), as well as in fish and other marine and terrestrial species (Dodder et al. 2002; Hale et al. 2002; Johnson and Olson 2001; Luross et al. 2002; Rice et al. 2002; Sellstrom et al. 1999; Voorspoels et al. 2003). Levels of PBDEs found in fish range from 180 ng/g lipid weight in salmon caught in the Baltic Sea (based on the sum of six tri-hexa-BDE congeners quantified) to 3,000 ng/g lipid weight in trout caught from Lake Michigan (Asplund et al. 1999; Manchester-Neesvig etal. 2001).

Human body burdens of PBDEs have increased markedly over the past several decades. For instance, Petreas et al. (2003) recently documented in samples of maternal serum that concentrations of BDE-47 in California women rose from below the detection limit to 50.6 ng/g lipid, comparing convenient samples of maternal serum collected between 1959 and 1967 with samples collected between 1996 and 1998. Other researchers have observed similar trends over the past three decades (Akutsu et al. 2003; Sjödin etal. 2004a).

The human health consequences of PBDE exposure have not been studied in detail. However, two reported adverse outcomes in laboratory animals dosed with high levels of PBDEs are neurologic deficiencies (Branchi et al. 2003; Eriksson et al. 2001; Viberg et al. 2003) and endocrine disruption (Rind 2002; Zhou etal. 2002).

Routes of human exposure to PBDEs are not fully defined. We hypothesized that a principal route of human exposure to PBDEs is through fish intake. This hypothesis is based on the fact that PBDEs are environmentally persistent and biomagnify in the marine food chain. Other potential routes of exposure such as inhalation may be of quantitatively greater importance than fish consumption, especially in the case of persons with occupational exposure (Sjödin et al. 2001). However, few studies have been conducted to evaluate the relative significance of these various routes of human exposure or to examine exposure source at different ages.

The purpose of this cross-sectional epidemiologic investigation was to determine whether consumption of fish caught in the Hudson River and Newark Bay by urban anglers was associated with increased body burden of PBDEs.

## Materials and Methods

### Study design and subjects.

During the summers of 2001–2003, a cross-sectional study was conducted among New York and New Jersey urban anglers ≥18 years of age. A total of 191 anglers were recruited with the aim to measure the association between body burdens of PCBs, mercury, and other organochlorines and reported fish intake. Participants recruited from local piers and fishing clubs provided questionnaire data, and 65% provided blood samples. A subsample of this population, 93 anglers with sufficient serum and complete questionnaire data, was selected for this analysis.

### Data collection.

After signed consent had been obtained, trained interviewers administered a questionnaire to each participant to obtain information on the frequency, species, and amount of locally caught fish consumed during the current fishing season. Respondents were asked to report the number of meals eaten per month as well as the usual serving size of the following species: American eel, black fish, blue crabs, blue fish, clams or mussels, flounder, fluke, striped bass, tommy cod, weak fish, white catfish, and/or white perch. Along with information regarding fish intake, demographic information and data on knowledge of fish advisories and practices related to cooking and cleaning fish were also obtained.

In addition to the questionnaire, participants were asked to provide a venous blood sample. Using venipuncture, a trained phlebotomist collected blood samples into 10-mL red-top Vacutainer tubes at the piers. Samples were centrifuged on site; serum was then processed and stored (at –20°C) at Mount Sinai until transported to the Centers for Disease Control and Prevention for analysis.

### Serum analyses for PBDEs.

The methodology used for the analysis of serum samples for PBDEs has been described in full elsewhere (Sjödin et al. 2004b) and is described briefly below. Samples were fortified with ^13^C-labeled internal standards, and formic acid was added to the samples as a denaturant; samples were finally diluted with water before solid-phase extraction (SPE). Liquid handling before extraction was automated using the 215 Liquid Handler (Gilson Inc., Middleton, WI), and SPE was automated using the Rapid Trace SPE workstation (Zymark, Hopkinton, MA). Removal of coextracted lipids was performed on a two-layered cleanup cartridge packed in an SPE tube, containing silica (top layer) and a mixture of silica sulfuric acid (bottom layer), using the SPE workstation for automation. Final analytical measurement of the target analytes was performed by gas chromatography–isotope dilution–high-resolution mass spectrometry using an MAT95XP instrument (ThermoFinnigan MAT, Bremen, Germany).

Concentrations of target analytes were calculated as nanograms per gram fresh weight (weight of serum) and nanograms per gram lipid weight (weight of serum lipids). Serum concentrations of total triglycerides and total cholesterol were determined using commercially available test kits (product nos. 011002803-0600 and 011573303-0600, respectively) from Roche Diagnostics Corp. (Indianapolis, IN) and a Hitachi 912 chemistry analyzer (Hitachi, Tokyo, Japan). All concentration data were corrected for analytical background, if detected, in method blank samples processed at the same time as the unknown samples. Blank samples (*n* = 3) and quality assurance/quality control samples (*n* = 3) were processed at the same time as the unknown samples. We conducted analyses for the following PBDE congeners (by International Union of Pure and Applied Chemistry numbers): BDE-47, BDE-85, BDE-99, BDE-100, BDE-153, BDE-154, and BDE-183. We could not report data for deca-BDE (BDE-209) because of high background contamination during the processing of the unknown samples. Results for PCB-153 are presented for comparison.

The limit of detection (LOD) when no analytical background was detected in blank samples was defined as a signal-to-noise ratio > 10. When an analytical background was detected in the blanks, the LOD was defined as three times the SD of the blanks.

### Data analysis.

Consumption of local fish by respondents was measured based on self-reported intake. Anglers who reported eating none of the fish species were categorized as “eating no locally caught fish”; this group was used as the reference group in all comparisons. Those respondents who reported eating any species at any frequency or amount were categorized in the statistical analyses as “eating locally caught fish.” Lipid-adjusted and non-lipid-adjusted geometric mean (GM) concentrations of the individual PBDE congeners were calculated and stratified by fish consumption category (any vs. none). In addition, total weekly fish consumption was calculated by summing species specific intake, and the following codes were assigned: no meals per month = 0, < 1 month = 0.05, 1 month = 0.25, 2–3 times per month = 0.50, 1 per week = 1, 2–3 times per week = 2.5, 4–5 times per week = 4.5, and ≥6 times per week = 6. Individuals were then categorized based on their total weekly fish consumption as having eaten locally caught fish *a*) never, *b*) less than once a week, or *c*) once or more a week. Associations between PBDE congeners and other covariates were not observed (data not shown). We calculated GMs and estimated differences between means and *p*-values using generalized linear models. All analyses were conducted using SAS software (version 8.2 for Windows; SAS Institute Inc., Cary, NC). Samples having concentrations < LOD were coded with the LOD.

## Results

Characteristics of the urban anglers and their self-reported frequency of fish intake are presented in Table 1. They were predominantly male (84%) and in their 50s, and they were racially diverse. Fifteen percent of the respondents reported no intake of locally caught fish. Anglers who reported not eating their local catch were mostly white and African American. More than 60% of this group reported having attended at least some college, and their annual incomes were evenly distributed. Among Hispanic, African-American, and Asian anglers, a larger proportion of anglers reported eating locally caught fish. The proportion of this group who reported having attending college was 46.3%, and roughly 50% reported annual household incomes < $50,000/year. Those anglers who reported not eating locally caught fish were heavier than those who ate local fish [body mass index (BMI) = 32.3 vs. 29.4]. The most commonly eaten species were fluke (76.3%) and striped bass (73.8%).

Data on serum PBDE concentrations by congener in the entire population studied are presented in Table 2; both unadjusted and lipid-weight-adjusted GMs, along with the minimum and maximum values, are given. The congener found at the highest concentration was 2,2′,4,4′-tetra-BDE (BDE-47; 0.091 ng/g fresh weight; 13.3 ng/g lipid weight). The next highest in concentration were 2,2′,4,4′,5-penta-BDE (BDE-99; 3.2 ng/g lipid weight); 2,2′,4,4′,5,5′-hexa-BDE (BDE-153; 3.2 ng/g lipid weight), and 2,2′,4,4′,6-penta-BDE (BDE-100; 2.7 ng/g lipid weight). For all other congeners (BDE-85, BDE-154, and BDE-183), GM concentrations were ≤1 ng/g lipid weight. BDE-209 was < LOD for all samples analyzed because of laboratory background. For BDE-85, BDE-154, and BDE-183, > 70% of the samples were < LOD, and for BDE-47 and BDE-153, < 10% of the samples were < LOD. Mean concentrations of PCB-153 were substantially higher than concentrations of PBDE congeners.

Geometric mean concentrations of the PBDE congeners stratified by reported local fish intake are shown in Table 3. For all PBDE congeners, GM concentrations were higher for anglers who reported eating locally caught fish than for anglers who reported not eating locally caught fish. However, differences were small and not statistically significant at an α-level of 0.05 for all congeners except BDE-183, for which 70% of samples were measured < LOD. For instance, anglers who reported intake of locally caught fish had a GM BDE-47 concentration of 13.4 ng/g lipid [geometric SD (GSD) = 3.3] compared with anglers who reported no intake (GM concentration, 12.6 ng/g lipid; GSD = 5.4). Concentrations in anglers who reported local fish intake are higher for all congeners; however, the greatest differences were observed for BDE-153, BDE-85, and BDE-183, where 70%, 59%, and 48% greater concentrations, respectively, were observed in anglers who reported local fish intake. The mean concentration of PCB-153 did not increase with reported fish intake.

Geometric mean serum levels for each PBDE congener are presented in Table 4, comparing anglers who reported no local fish consumption, fish intake once or more a week, and fish intake more than once a week. For BDE-100 and BDE-153, we observed a moderate increase in mean concentration across these three strata as reported fish consumption increased; this was also true for BDE-85 and BDE-183, where a greater proportion of the samples were measured < LOD. However, the mean concentrations of all congeners were higher among anglers who reported fish intake more than once a week compared with those who reported no intake. Mean concentration of PCB-153 was lowest among anglers who reported eating local fish once a week or less, with similar mean concentrations for those who reported eating no fish and those who reported eating locally caught fish more than once a week.

## Discussion

In the present study we found differences albeit small, in mean concentrations of PBDEs for anglers who reported eating locally caught fish compared with those who eat no local fish. These differences did not reach statistical significance at α= 0.05 for most of the congeners examined. Because congener-specific GMs were consistently higher for anglers who reported eating their catch and because dose–response patterns were observed for some congeners, the possibility cannot be excluded that consumption of locally caught fish may be a route of PBDE exposure. However, these data suggest that consumption of locally caught fish is certainly not a major route of exposure in this population.

Our findings differ from those reported by other investigators who have studied anglers. For example, fish consumption has been shown to be a major exposure route in Swedish fishermen consuming large quantities of Baltic Sea fish (Sjödin et al. 2000). This Swedish study found that people reporting high fish consumption had a median BDE-47 level five times higher [2.2 ng/g lipid weight; 10 and 90% confidence interval (CI), 0.96–5.7] than that of nonconsumers (0.4 ng/g lipid weight; 10 and 90% CI, < 0.1–2.5).

The null effects observed in the present study may be due to a variety of factors. First, our data were limited by large variability in the levels of each congener. For instance, the measured lipid-adjusted concentrations of BDE-47 ranged from 0.71 to 1,389 ng/g lipid weight, with a median of 10.8 ng/g lipid weight. Although the data were normalized in the analyses using GMs, substantial variation remained within fish eaters and noneaters, making the ability to observe differences between groups difficult. This issue of variation may be resolved in future studies by increasing the sample size. In addition, other factors have influenced our ability to detect differences such as the quantity of the samples. Although variation in the chemical measurements remained low (quality assurance/quality control, 4.9–7.0%), substantial LOD variation remained within congeners. This may be improved in future studies with greater and similar serum samples for each participant.

Other unmeasured exposure routes to PBDEs may also have affected our ability to detect associations. For instance, a recent analysis of indoor dust samples collected in the United States and Germany has suggested that significantly higher levels of tetra-BDE and deca-BDE congeners are found in the United States compared with Germany. The median levels of BDE-47, BDE-99, BDE-153, and BDE-209 for the dust samples collected in the United States were 430 ng/g dust (range, 230–3,000 ng/g), 880 ng/g dust (range, 69–3,700 ng/g), 140 ng/g dust (range, 5–650 ng/g), and 2,000 ng/g dust (range, 120–21,000 ng/g), respectively. In Germany, by contrast, < 14 ng/g dust (range, < 14–22 ng/g), 10 ng/g dust (range, < 4–38 ng/g), < 6 ng/g dust (range, < 6–22 ng/g), and 60 ng/g dust (range, < 5–410 ng/g) were found for BDE-47, BDE-99, BDE-153, and BDE-209, respectively (Sjödin et al. 2004c). However, there is no reason to believe that differential indoor dust exposure exists between consumers and nonconsumers of locally caught fish. Nevertheless, exposure to indoor dust may be an exposure route for this population. It is possible, however, that there is a differential bias due to consumption of commercial fish. A recent market basket study has shown that commercial fish and shellfish have high levels of PBDEs (Bocio et al. 2003), although information on store-bought fish was not elicited during the interviews in the present study.

Although we did not observe statistically significant differences by reported fish consumption, serum concentrations of PBDEs in this study are similar to those reported in other populations. In the United States, Mazdai etal. (2003) reported a median concentration of BDE-47 of 28 ng/g lipid in maternal serum; Sjödin et al. (2004a) reported a mean concentration in archived serum pools of 34 ng/g lipid; and Schecter et al. (2003) reported a mean concentration in maternal breast milk of 40.8 ng/g lipid. The GM concentration of BDE-47 in our study was 13.3 ng/g lipid (median = 10.79 ng/g lipid weight), a somewhat lower concentration than findings from other U.S. populations. These levels are higher than concentrations reported in Europe, where, for instance, Lind etal. (2003) reported a mean concentration of BDE-47 of 2.35 ng/g lipid weight in breast milk for Swedish women sampled between 1996 and 1999.

Although the Great Lakes Chemical Company, a primary North American manufacturer of PBDEs, voluntarily agreed to stop producing octa- and penta-BDEs in 2004, the company will continue to produce deca-BDEs (Great Lakes Chemical Corporation 2003). Moreover, the persistent nature of these chemicals, in addition to the lack of understanding of how they degrade, leaves room for concern about human contamination. Future studies that continue to monitor human body burdens of PBDEs as well as define exposure pathways are needed to further our understanding of how these chemicals influence human health.

## Figures and Tables

**Table 1:** Characteristics of urban anglers and reported fish intake (*n* = 93).

Characteristic	No local fish intake (*n* = 14)	Any local fish intake (*n* = 79)
Male (%)	85.7	83.8
Age, years (mean ± SD)	58.5 ± 11.2	50.1 ± 14.0
Race (%)		
White	78.6	60.0
African American	14.3	18.8
Hispanic	7.1	16.3
Asian	0.0	2.5
Other/no response	0.0	2.5
Total yearly household income (%)		
< $10,000	7.1	8.8
$10,000–29,999	21.4	20.0
$30,000–49,999	21.4	22.5
$50,000–74,999	21.4	17.5
≥$75,000	21.4	15.0
Not reported	7.1	16.3
Highest level of school completed (%)		
< High school graduate	21.4	12.5
Graduated high school	14.3	41.3
≥Some college	64.3	46.3
BMI (mean ± SD)	32.3 ± 6.6	29.4 ± 4.9
Reported intake of any of the following species of locally caught fish (%)		
American eel		21.3
Black fish		43.0
Blue crab		42.5
Blue fish		65.0
Clams or mussels		20.3
Flounder		60.0
Fluke		76.3
Striped bass		73.8
Tommy cod		11.3
Weak fish		57.5
White catfish		8.8
White perch		13.8

**Table 2:** Mean concentration of PBDEs in human serum.

		Unadjusted (ng/g fresh weight)^a^			Lipid adjusted (ng/g lipid weight)^a^			< LOD
	No.^b^	GM	Minimum	Maximum	GM	Minimum	Maximum	(%)
PBDE congener								
47	93	0.091	0.005	12.613	13.288	0.706	1388.649	7
85	92	0.007	0.002	0.685	1.033	0.200	109.096	73
99	93	0.022	0.002	3.318	3.225	0.334	545.541	33
100	93	0.010	0.002	2.548	2.701	0.300	280.615	12
153	93	0.022	0.003	1.500	3.166	0.389	165.162	4
154	89	0.004	0.000	0.224	0.630	0.088	24.711	75
183	93	0.004	0.001	0.017	0.525	0.115	2.015	71
PCB-153	80	0.407	0.038	2.794	60.518	9.720	495.903	0

a Geometric minimum, and maximum.

b Number of participants.

**Table 3:** GM concentration of PBDEs by local fish intake (ng/g lipid weight).

	No local fish intake		Any local fish intake		
	No.^a^	GM (GSD)	No.^a^	GM (GSD)	*p*-Value
PBDE congener					
47	14	12.61 (5.42)	79	13.41 (3.30)	0.87
85	14	0.70 (3.56)	78	1.11 (3.54)	0.21
99	14	2.83 (4.69)	79	3.30 (3.24)	0.67
100	14	2.32 (4.66)	79	2.77 (2.94)	0.59
153	14	2.02 (4.13)	79	3.43 (2.88)	0.10
154	12	0.56 (3.74)	77	0.64 (2.09)	0.57
183	14	0.38 (1.99)	79	0.56 (1.65)	0.01
PCB-153	14	65.19 (2.25)	66	59.57 (2.30)	0.71

a Number of participants.

**Table 4:** GM concentration of PBDEs by frequency of reported local fish intake (ng/g lipid weight).

	No local fish intake		Fish intake ≤1/week		Fish intake > 1/week	
	No.^a^	GM (GSD)	No.^a^	GM (GSD)	No.^a^	GM (GSD)
PBDE congener						
47	14	12.61 (5.42)	25	11.55 (3.07)	54	14.37 (3.41)
85	14	0.70 (3.56)	25	0.89 (3.28)	53	1.23 (3.65)
99	14	2.83 (4.69)	25	2.68 (2.92)	54	3.63 (3.38)
100	14	2.32 (4.66)	25	2.34 (2.63)	54	3.00 (3.08)
153	14	2.02 (4.13)	25	2.58 (3.06)	54	3.91 (2.76)
154	12	0.56 (3.74)	23	0.51 (1.91)	54	0.71 (2.13)
183	14	0.38 (1.99)	25	0.49 (1.70)	54	0.59 (1.62)
PCB-153	14	65.19 (2.25)	20	40.58 (2.06)	46	70.40 (2.29)

a Number of participants.
